# Factors of customers’ channel choice in an omnichannel environment: a systematic literature review

**DOI:** 10.1007/s11301-022-00281-w

**Published:** 2022-07-21

**Authors:** Lukas Wolf, Martina Steul-Fischer

**Affiliations:** Friedrich-Alexander-Universität Erlangen-Nürnberg (FAU), Lange Gasse 20, 90403 Nuremberg, Germany

**Keywords:** Channel choice, Omnichannel management, Multichannel management, Consumer behavior, Customer behavior, Systematic literature review, M310, M370, O330

## Abstract

The proliferation of mobile devices and the continuous development of online technologies has led to an increasing variety of channels, leaving customers with a choice of channel alongside the choice of product, service, or retailer. Any attempt to optimize customer experience and engage in successful omnichannel management will require a complete, multifaceted understanding of the processes around channel choice of customers. To date, the many existing studies around multi- and omnichannel research have failed to yield an integrated, comprehensive synthesis of factors involved in customers´ channel choice. Our study conducted a systematic literature review to the end of identifying the factors involved in channel choice which appear in the scientific literature on this topic over the last two decades. We retrieved 128 papers from three bibliographic databases (EBSCO Host, Scopus, and Web of Science) and carried out descriptive analysis on them. Qualitative thematic analysis inductively extracted 66 different factors of channel choice, each assignable to five broader categories, from the studies included in the review. The findings indicate that perceived channel characteristics, customer needs and situational or contextual factors influence customers´ channel choice directly, and customer characteristics and characteristics of products or services influence it indirectly. Alongside its presentation of an integrated conceptual framework comprising these relationships, our study details a comprehensive research agenda with regards to theories, contexts, and methods and, in particular, with regards to factors influencing customers´ channel choice. Our findings advance the academic understanding of channel choice behavior and provide researchers and practitioners in this area with information on important implications for omnichannel management.

## Introduction

The proliferation of mobile devices and the ongoing development of online technologies has led to a constantly increasing variety of channels, such as mobile apps and social media (Li et al. 2017). This leaves today’s customers with the choice of channel as an additional factor alongside their choice of product, service, and retailer (Xu and Jackson 2019a). Understanding the factors involved in channel choice from a customer perspective has always been an important part of channel management (Neslin et al. 2006). The recent move toward synergetic management of multiple channels in the context of omnichannel management places customer orientation still higher on the agenda (Lemon and Verhoef 2016; Verhoef et al. 2015). Numerous studies (for example Saghiri et al. 2017; Wagner et al. 2020) have emphasized the importance of understanding the factors driving channel choice to the enhancement of customer experience and the consequent improvement of omnichannel management. The multitude of studies around multi- and omnichannel research driven by this exigency (see, for example, Barwitz and Maas 2018; Gensler et al. 2012), have yet failed to yield an integrated, comprehensive synthesis of factors in customers´ channel choice, despite some initial attempts at summaries (e.g. Li et al. 2017; Neslin et al. 2006). This paper will therefore seek to advance our understanding of channel choice behavior in an omnichannel environment by identifying factors of channel choice and synthesizing the existing body of knowledge via a systematic literature review (SLR).

The key principles of an omnichannel environment include: (1.) the offering of merchandise and services through a multitude of available channels, (2.) integration of channels into a unified system from a retailer´s point of view (i.e. enabling the same task-fulfillment on every channel), and (3.) seamless inter-channel interaction from a customer perspective (i.e. allowing for easy switching between channels or devices along the customer journey) (Beck and Rygl 2015; Verhoef et al. 2015). This means that, successful omnichannel strategies require an understanding of both the retailer´s and the customer´s perspective. This notwithstanding, most conceptual studies and SLRs in this area to date address multi- or omnichannel related topics solely from a retailer or management perspective (cf. Appendix A). For instance, Gao et al. (2020) performed a literature review to the end of systematically summarizing studies on multichannel integration along the customer journey. Hossain et al. (2019) examined multichannel integration quality within service delivery channels with the aid of a SLR and qualitative interviews. Gerea et al. (2021) summarized and synthesized the existing body of research on omnichannel customer experience management highlighting the importance to omnichannel businesses of pursuing a customer-centered approach. Wang et al. (2021b) reviewed current studies from the research fields of information systems, operations and marketing, presenting a multidisciplinary view of omnichannel retailing. Finally, Cai and Lo (2020), Lopes et al. (2021), and Salvietti et al. (2021) made use of bibliometric approaches such as bibliographic modeling via citation analysis to aggregate findings on omnichannel management and to propose research fields in this domain. The only SLR to date that takes a customer perspective is Mishra et al. (2021), which explored the cognitive, affective, and conative dimensions of general consumer decision-making in omnichannel retailing. This review´s authors outlined the rapidly evolving research within this field and emphasized the importance of investigating customers´ channel choice behaviors in a separate study (Mishra et al. 2021).^1^ The research presented here differs from the SLR by Mishra et al. (2021) in its intent of establishing factors of customers’ channel choice in a multi- and omnichannel environment, rather than setting out a general examination of customer behavior in omnichannel environments. The criteria for the selection of literature for the review, including keywords, databases, and time span, and the descriptive and thematic analysis conducted diverge from those in Mishra et al. (2021).

The existing body of research on channel choice in multi- and omnichannel environments encompasses a diverse range of heterogeneous studies, conceptual papers, and other types of scientific work; we can therefore consider this topic mature in research terms, which means it is eminently suitable for a thorough SLR that both synthesizes and expands it (Paul et al. 2021; Webster and Watson 2002). However, the plethora of SLRs to date on multi- or omnichannel related topics have been unable to draw conclusions on customers’ behaviors as regards channel choice. The present study is therefore one of the first reviews on multi- and omnichannel management to proceed from a customer perspective and the first attempt to systemically summarize and conceptualize factors of customers´ channel choice in this context. It therefore contributes to the literature on multi- and omnichannel environments in multiple ways: First, in synthesizing and categorizing factors of channel choice, the review advances our understanding of customer behavior, which is vital to successful omnichannel management (Mishra et al. 2021; Verhoef et al. 2015). The inductive approach taken by the thematic content analysis described in this article yields a conceptual framework for use by researchers and practitioners as an aid to their comprehensive understanding of customers’ channel choice. Second, the article, drawing on a descriptive and thematic analysis of relevant published work, sets out an extensive research agenda encompassing contexts, theories, methods, and particularly factors of channel choice for research in this area. Finally, it delineates a number of implications for channel management.

The findings of our literature review indicate that customers´ channel choice behavior is a highly complex process influenced by a variety of factors, including channel characteristics, customer needs, and situational or contextual factors. While some indirectly influential factors of channel choice, such as customers´ age are frequently the subjects of academic study, the current literature in the area of multi- and omnichannel business lacks understanding of the directly influential factors. Further, extant channel choice research concerns itself with a limited number of industries or products and countries, and its findings require validation via the introduction of other methods (e.g. longitudinal studies) and research designs and via the use of theoretical foundations and models.

The review is structured as follows. After discussing the methodology used for the SLR, the paper provides a descriptive overview of the articles retrieved for the literature review and then proceeds to analyze factors of channel choice as represented in the literature identified. Following this, a section proposing potential areas for future research on channel choice behaviors sets out a comprehensive research agenda. The concluding section points to implications and limitations of this study.

## Methodology

The relevance of SLRs to research in the area of business is currently higher than it has ever been (Snyder 2019). In collating and evaluating findings from numerous empirical studies, SLRs provide an overview of heterogeneous and interdisciplinary research domains and help prevent bias by bringing together evidence at a meta-level (Snyder 2019). They can also act as foundations or springboards for future research or apply a particular perspective to it (Paul and Criado 2020). The basis of a SLR may be either a research domain, a theory or a research methodology (Paul et al. 2021; Paul and Criado 2020). The present review falls into the domain-based category; more specifically, it is a structured theme-based review (Paul et al. 2021) conducted to the end of providing an overview and synthesis of the determining factors of channel choice, from a customer perspective, identified within the existing literature on multi- and omnichannel retail. This approach was most conducive to our objectives, as it enabled us to develop a thorough understanding of the literature in the area of channel choice and identify relevant gaps and, ultimately, a future research agenda.

Many guidelines on conducting SLRs recommend the development of a detailed systematic review protocol to ensure transparency and reproducibility of the review process (Kitchenham and Charters 2007; Okoli 2015; Paul et al. 2021; Snyder 2019). We accordingly determined our search strategy prior to carrying out the SLR; this entailed the selection of search terms, relevant databases, and inclusion criteria (Fisch and Block 2018; Paul et al. 2021).

*Search terms and relevant databases:* A search string requires the use of words and phrases (i.e. search terms) directly related to the research question (Snyder 2019). This presented us with a challenge, as academic publications do not demonstrate a consistently uniform understanding or use of the terms “multi- “, “cross- “, or “omni-channel” (Beck and Rygl 2015). Nor is there a universally accepted definition of “channel choice” as a concept; other terms used to describe it include “interaction choice” (Barwitz and Maas 2018), “channel preference” (Becker et al. 2017; Boardman and McCormick 2018), “adoption of channels” (Bilgicer et al. 2015), or “channel use/usage” (Frasquet et al. 2015, 2019). We therefore sought to prevent exclusion of relevant results by testing a number of alternative terms, synonyms, and abbreviations of “choice” and “omnichannel”. The final search string, constructed with the aid of Boolean operators and truncations, included the terms: (choice OR choose* OR select* OR use OR usage OR utili* OR adopt* OR prefer*) AND ("omni channel" OR "multi channel" OR "cross channel" OR "dual channel" OR omnichannel OR multichannel OR cross-channel OR dualchannel). To the end of comprehensively covering the relevant literature, we searched within three electronic literature databases: EBSCO Host, Scopus, and Web of Science (Kuckertz and Block 2021; Wanyama et al. 2021). These databases are considered the most important and widely used within research in business and the social sciences and consequently find frequent use for SLRs within the business field (examples are Eckert and Hüsig 2022; Lu et al. 2018b; Neuhaus et al. 2021; Tueanrat et al. 2021).

*Inclusion criteria:* The inclusion criteria we defined for our review related to language, year of publication, source type, domain, journal quality, and an assessment of the content and research design of the article in question (Kitchenham and Charters 2007; Okoli 2015; Paul et al. 2021; Paul and Criado 2020; Snyder 2019). To meet the criteria for inclusion, papers had to be written in English and published after 2000 in peer-reviewed journals from the business discipline with an impact factor greater than 1 (Paul et al. 2021).^2^ We set the beginning of the time frame for the search at the year 2000 due to the rarity of commercialized online channels and therefore of research on multichannel or omnichannel management prior to the turn of the millennium (Gao et al. 2020; Hossain et al. 2019). For the content assessment, two researchers independently read the abstracts of all articles after the initial identification (n = 924) and judged whether they addressed channel choice from a customer perspective. There was agreement between the researchers on suitability or non-suitability in this regard for 94.48% of the articles (n = 873). To further ensure validity and reliability, a third researcher conducted an independent assessment of the articles upon which the initial two researchers did not agree (n = 51).

**Fig. 1:**
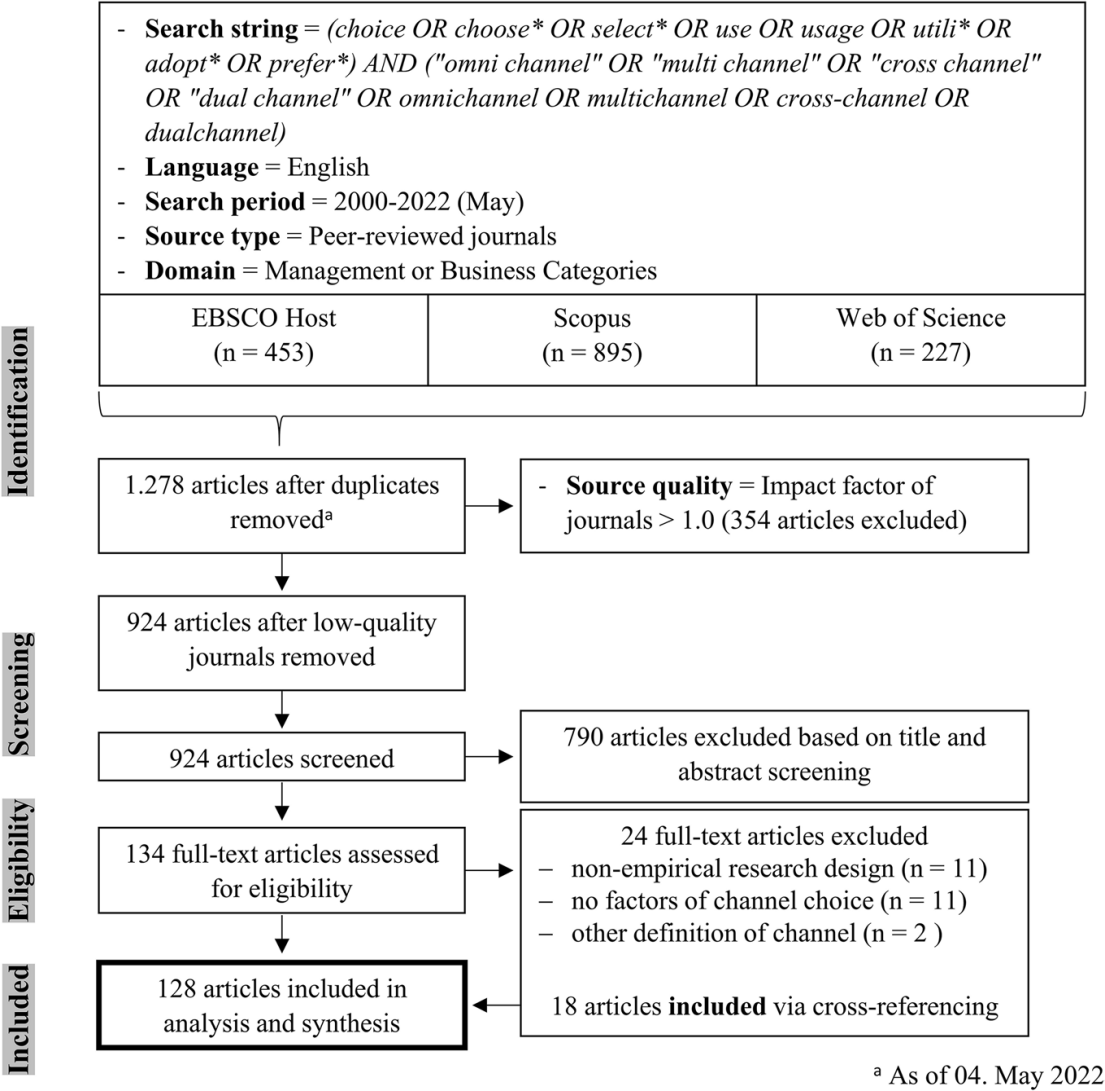
The search process based on PRISMA guidelines (Moher et al. 2009)

The papers selected at this stage (n = 134) were read in detail and assessed for content and method eligibility. This process resulted in the exclusion of thirteen studies due to inapposite content, mainly because the articles examined the consequences of channel choice rather than the reasons behind it (one example is Herhausen et al. 2019). We also removed eleven non-empirical studies, such as introductions to special issues of journals (e.g. Thaichon et al. 2022). Cross-referencing yielded 18 further articles that were eligible for inclusion (see Appendix B). The final selection of published research on channel choice comprised 128 papers. Figure 1, developed following the PRISMA guidelines (Moher et al. 2009), provides an overview of the complete search process. Appendix B lists the articles included in the final review.

*Extraction of data and analysis:* The articles ultimately selected for the review were synthesized and structured with the aid of a concept matrix (Webster and Watson 2002). First, we extracted descriptive information from each article, such as year of publication, research focus (omnichannel, multichannel, or both), channels considered, research contexts (country, industry), customer journey stage investigated (i.e. pre-purchase, purchase or post-purchase) and methodology employed (Snyder 2019). We used Microsoft Excel for the concept matrix and the subsequent descriptive analysis. For the thematic analysis, we followed the widely-used qualitative process proposed by Braun and Clarke (2006). This process entails familiarization with the data (i.e. the articles retrieved), generating initial codes with regards to interesting features, searching for themes across these codes, reviewing and refining these themes with the aid of a thematic map and, finally, defining and naming the themes and producing the report. In line with this process, we inductively coded factors of channel choice using the MAXQDA software and generated five categories/themes on the basis of the literature review, including 14 sub-categories/sub-themes and 66 factors/codes of channel choice.

## Profile of the literature on channel choice

**Fig. 2:**
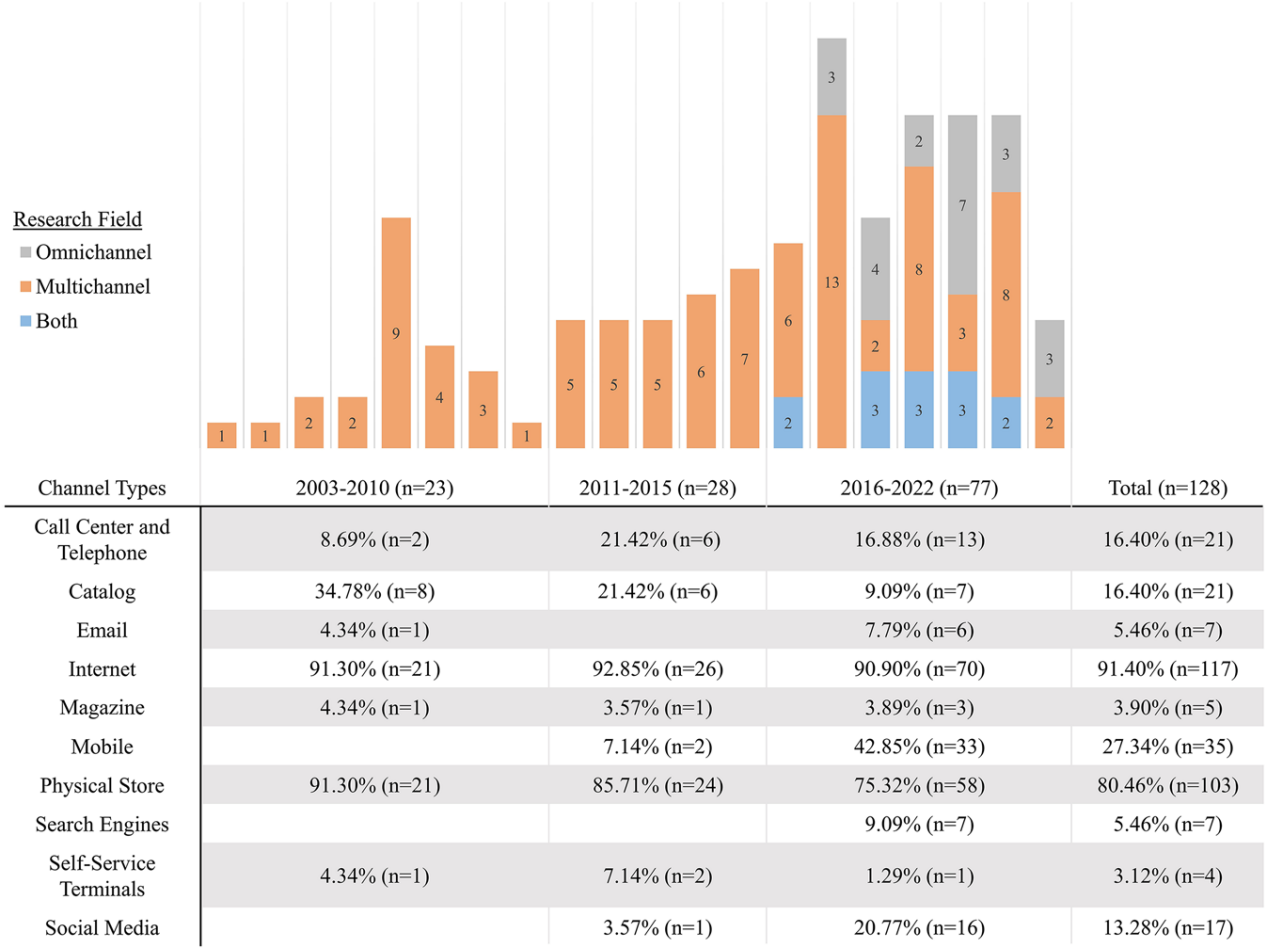
Distribution of channel choice articles across years of publication and research fields. *Note*: The list of channels is not complete, as we included only the ten channels most frequently mentioned in the literature. Among the channels omitted here are agencies (e.g. Hosseini et al. 2018) and internet-enabled TVs (e.g. Wagner et al. 2020). The “internet” channel includes all online channels investigated in the studies which were not specified further (examples include websites, price comparison sites, online shops). “Mobile” channels are cited as having occurred in articles which considered the online channel for mobile devices separately from the static online channel (e.g. Sands et al. 2016). As most studies on channel choice investigate two or more different channels, the totals exceed the number of articles retrieved for the review

Exploring channel choice from a customer perspective has always been an integral part of channel research (Neslin et al. 2006). However, with the proliferation of mobile devices and new technologies, and the increasing prevalence of omnichannel management, the understanding of channel choice has become all the more important in recent years (Barwitz and Maas 2018). This trend is confirmed by our review. As Fig. 2 illustrates, the number of articles published that met our selection criteria has more than doubled in the past six years. Of the 128 papers retrieved, 77 (~ 60%) were published between 2016 and May 2022; of these, 35 (~ 45%) explicitly address omnichannel management. Contrastingly, all articles selected that appeared prior to 2016 focus exclusively on channel choice in a multichannel environment. Most studies consider the traditional internet channel or physical stores in their investigation of channel choice (91% and 80% respectively of the articles retrieved). In recent years, emerging online channels such as mobile channels (i.e. the accessing of online channels via mobile devices), social media, and search engines have gained a greater share of attention, and, in general, the variety of channels considered has diversified in the last years in line with the move towards an omnichannel environment. Only a few channels, such as physical catalogs, have received less attention in the course of time. This finding is consistent with Konuş et al. (2014).

We identified 58 journals that published papers related to channel choice within multi- or omnichannel environments in the last two decades. The highest number of articles were published by the *Journal of Retailing and Consumer Services* (n = 23), followed by the *International Journal of Retail and Distribution Management* (n = 12) and the *Journal of Business Research* (n = 8). Most of the studies do not indicate the application of specific theories to explain customers´ channel choice. However, some studies are explicitly grounded in popular theories of consumer and technology research, such as the theory of planned behavior (e.g. Pookulangara et al. 2011a), the theory of reasoned action (e.g. Pookulangara et al. 2011b) and the diffusions of innovations theory (e.g. Bilgicer et al. 2015).

Table 1 shows the distribution of the articles across research contexts. Of the 128 articles retrieved, 114 explicitly state the country in which the research was conducted and 105 the relevant industrial context, while 118 indicated the stage of the customer journey that the work observed. Over a quarter of the studies (n = 34) examined channel choice in the U.S. The distribution across sectors is more diverse. The most frequently studied industrial context is fashion and beauty (n = 36), including clothing (e.g. Lu and Rucker 2006) and cosmetics (e.g. Chiou et al. 2017); consumer electronics follow in second place (n = 20) and financial services and insurances in third place (n = 17). In terms of the customer journey stage investigated, most studies (n = 49) focused on channel choice for a combination of the pre-purchase stage (i.e. recognition of needs, consideration, search) and the purchase stage (i.e. choice, ordering, payment). Several studies examined channel switching between these stages (phenomena such as “showrooming” for switching from offline to online – an example is Daunt and Harris 2017 – and “webrooming” for switching from online to offline, e.g. Santos and Gonçalves 2019). Seven studies analyzed the post-purchase period (i.e. consumption, usage, engagement, service requests) separately, mostly in terms of the choice of channel for making a complaint (e.g. Miquel-Romero et al. 2020).

**Table 1:** The most frequently occurring contexts within the channel choice articles retrieved for the review

	Country	No. of articles	Sector	No. of articles	Stage of customer journey	No. of articles
1	United States	34	Fashion and beauty	36	Pre-purchase and purchase	49
2	China	13	Consumer electronics	20	Purchase	36
3	Spain	10	Financial services and insurance	17	All stages	21
4	United Kingdom	10	Travel and tourism	8	Post-purchase	7
5	Germany	9	Groceries	7	Purchase and post-purchase	2
			Books and CDs	7	Pre-purchase	2

For the most part, the studies are quantitative (n = 110, ~ 86%), with surveys and company database analysis the most commonly used methods (Table 2). Qualitative methods such as interviews and focus groups make relatively rare appearances, as do mixed methods approaches, used by only five articles (for example Mahrous and Hassan 2017).

**Table 2:** Research design and methods within the channel choice articles retrieved for the review

Research design	No. of articles	Method	No. of articles
Quantitative	110	Survey	85
Qualitative	13	Company database	29
Mixed methods	5	Interview	14
		Experiment	12
		Focus group	9

As some articles use more than one method (as an example, Schröder and Zaharia (2008) use focus groups, face-to-face interviews and surveys), the totals exceed the number of articles retrieved

## Analysis and findings

### Factors of channel choice

This section summarizes the factors of channel choice identified within the articles reviewed. In total, we extracted 66 distinct factors from the studies and grouped them into 14 subcategories and five main categories (see Tables 3–7).

#### Perceived channel characteristics

Customers tend to choose channels that are able to fulfill their needs in a convenient, low-risk and low-cost manner (Schröder and Zaharia 2008; Sonderegger-Wakolbinger and Stummer 2015). They weigh the channel’s perceived benefits against its perceived drawbacks (Harris et al. 2017; Singh and Jang 2022). Within the concept of “channel benefits,” we can distinguish the notions of “channel quality” and “channel convenience,” while “channel risks” and “channel costs,” by contrast, are typical disadvantages of a specific channel (Gensler et al. 2012) (see Table 3).

**Table 3:**
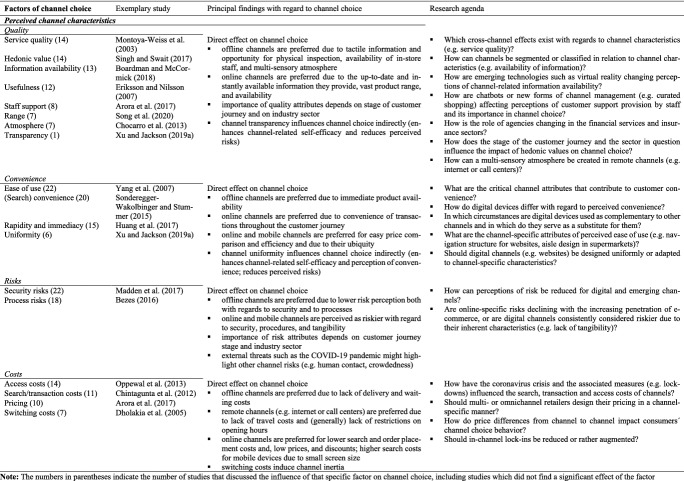
Factors of channel choice investigated in the articles reviewed – 1. Perceived channel characteristics

*Channel quality* relates to the channel’s perceived ability to fulfill the needs and expectations of customers (Gensler et al. 2012; Oppewal et al. 2013). Gensler et al. (2012) identify channel quality as the most important channel attribute at the pre-purchase and purchase stages. The studies discuss, *inter alia*, quality of customer service provided, hedonic aspects, availability of information, usefulness, customer care and support provided by staff, the range of products or services available, and atmosphere as influential channel quality attributes. While support from staff and atmosphere are often considered benefits specific to offline channels (see, for example, Arora et al. 2017), range variety and availability are more online-specific advantages (e.g. Boardman and McCormick 2018). The influence of perceived availability of information through a specific channel depends on the type of information needed. Brick-and-mortar stores are particularly well suited to providing tactile information and permit the potential customer to inspect the product physically (Dholakia et al. 2005). Internet channels, by contrast, stand out for the infinite access they provide to up-to-date information from a variety of sources (Cheng and Huang 2014; van Nguyen et al. 2022). Telephone contact and call centers, meanwhile, offer fast, personalized situational support and information (Jerath et al. 2015). Quality of customer service and channel usefulness are significant factors in channel choice across all channels (see, for example, Eriksson and Nilsson 2007; Frasquet et al. 2015; Montoya-Weiss et al. 2003; Yang et al., 2007). Hedonic aspects tend to play a minor role in channel choice (Hussein and Kais 2021; Singh and Swait 2017).

*Channel convenience* can be defined as the perceived ease and speed with which a customer can use the channel along the customer journey (Gensler et al. 2012). We identified four factors relating to channel convenience within the articles retrieved: perceived ease of use, search-related convenience, rapidity and immediacy, and channel uniformity. Online channels appear particularly convenient at the pre-purchase stage (Sonderegger-Wakolbinger and Stummer 2015), and customers value the usability and user-friendliness of online and mobile channels, particularly with regard to price comparison (Boardman and McCormick 2018; Singh and Swait 2017) and efficiency (Li et al., 2019). Mobile channels seem to perform very well in terms of rapidity and immediacy due to the portable and ubiquitous nature of mobile devices (Huang et al. 2017) and the resulting lack of spatial or temporal constraints on the shopping experience (Kim et al. 2019; Rodríguez-Torrico et al. 2017). A major benefit of brick-and-mortar stores, meanwhile, is immediate product availability (Boardman and McCormick 2018), meaning physical stores largely win for impulse shopping and urgent purchasing needs (Harris et al. 2017; Kazancoglu and Aydin 2018). Wang et al. (2016) indicate that the significant influence of perceived convenience is limited to attitudes toward using physical channels and does not come into play in relation to online channel use at the purchase stage. This may be because other attributes, such as perceived channel risks, eclipse customer perceptions of convenience for the online channel in this phase of the customer journey. Xu and Jackson (2019a) lend support to this hypothesis, having found that channel convenience positively influences perceived channel-related customer self-efficacy, but has no significant effect on perceived risk.

The perception of *channel risks* appears most prominent in connection with online and mobile channels (see, for example, Bezes 2016; Kollmann et al. 2012). This is because some perceived risks are particularly pronounced in online and mobile shopping, namely security risks (concerns around the safety of user and payment information), procedural risks (concerns about the shopping process), and intangibility risks (concerns resulting from a lack of immediate gratification and tangibility) (Lee and Jung 2020). The brick-and-mortar store environment facilitates customers’ immediate and generally reassuring assessment of these risks. Accordingly, perceived risks of offline channels, such as privacy or security concerns, appear to have no significant effect on attitudes toward purchasing via offline channels (Eckl and Lingenfelder 2021; Wang et al. 2016). The perception of risks relating to online channels, by contrast, results directly in a decrease in online channel use (Lee and Jung 2020; Madden et al. 2017; Montoya-Weiss et al. 2003) and greater use of alternative channels such as brick-and-mortar stores (Chiu et al. 2011; Kukar-Kinney and Close 2010). However, arising from the COVID-19 pandemic and associated threats such as danger of infection, other channel risks like human contact gain importance and consumers´ channel choice becomes health-related behavior (Eckl and Lingenfelder 2021; Wang et al. 2021a). For instance, Wang et al. (2021a) showed that perceived susceptibility to COVID-19 combined with the perceived severity of the virus positively influences the value of channels that do not involve human contact.

*Channel costs* may be monetary or non-monetary. Customers tend to consider costs in time, money and effort for access to or the use of channels to be lower for channels that can be accessed remotely (Lipowski and Bondos 2018). For instance, expense of time and money on traveling, which is influential in channel choice (Chocarro et al. 2013; Oppewal et al. 2013; Sousa et al. 2015), is generally not a factor in use of the internet or call centers (Venkatesan et al. 2007). Accordingly, Kollmann et al. (2012) find that online channels cannibalize brick-and-mortar stores if customers perceive the latter as being too far away. However, online shopping might entail delivery costs or costs of waiting for the delivery to arrive (Chintagunta et al. 2012). Switching costs may result from implicit or explicit in-channel lock-ins including incompatibility between channels, requiring the customer to learn the new channel’s structure (Dholakia et al. 2005), low levels of channel integration (Shen et al. 2018), and satisfaction with the current channel (Falk et al. 2007). Sonderegger-Wakolbinger and Stummer (2015) found that the opportunity to switch is a determining factor of channel choice along the customer journey. In addition to lower channel costs, numerous studies have shown that online channels in particular are associated with special offers and bargains (Huang et al. 2017), which may lead price-conscious customers to prefer them at the purchasing stage (Arora et al. 2017; Schneider and Zielke 2020).

#### Customer needs

The relative importance of channel characteristics to a customer depends in large part on that customer’s needs.^3^ For instance, customers whose needs for customer service or comfort prompt a wish to interact with sales staff will tend to visit a physical store (Kazancoglu and Aydin 2018). If they want to compare prices easily (i.e. convenience-seeking) or require a large range (i.e. variety seeking), they will tend to use online channels (Boardman and McCormick 2018; Cervellon et al. 2015; Santos and Gonçalves 2019).

We find two general types of customer needs within the channel choice literature: utilitarian and hedonic needs (see, for example, Blázquez 2014; Boardman and McCormick 2018; Cervellon et al. 2015; Heitz-Spahn 2013; Pookulangara et al. 2011b) (see Table 4). *Utilitarian needs* include rationally-driven customer requirements for an improved utility of the customer experience, such as savings in time or money. Such needs appear as a means to an end and are primarily cognitive, functional and instrumental in character (Babin et al. 1994; Childers et al. 2001). *Hedonic needs* relate to the potential entertainment and enjoyment resulting from the shopping experience and thus are more affective and multi-sensory (Childers et al. 2001; Holbrook and Hirschman 1982). With regard to channel choice, we classify needs such as the need for information or customer service, convenience-seeking, the need for touch, cost avoidance, the need for comfort and redress-seeking as utilitarian needs, and enjoyment-seeking, variety-seeking, purchase proneness, and sustainability orientation as hedonic needs. Several studies suggest that the influence of utilitarian needs on channel choice is greater than that of hedonic needs (Blázquez 2014; Cervellon et al. 2015; Hallikainen et al. 2019; Pookulangara et al. 2011a).

**Table 4:**
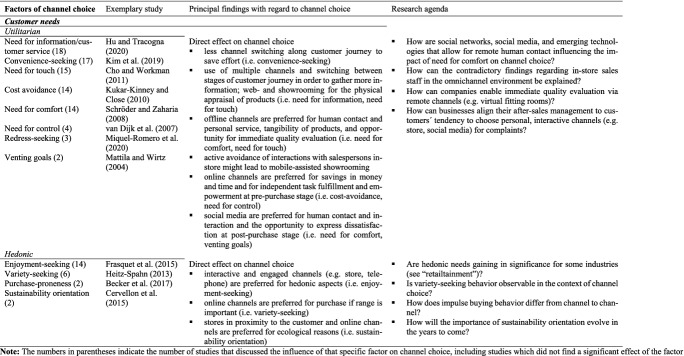
Factors of channel choice investigated in the articles reviewed – 2. Customer needs

A need for information leads to the use of multiple channels for searching (Hu and Tracogna 2020; van Nguyen et al. 2022) and the switching of channels between pre-purchase and purchase (webrooming and showrooming; Maggioni et al. 2020; Sands et al. 2016), driven by the customer’s wish to gather as much information as possible by making use of various sources. Convenience-seeking explains the use of mobile channels for the pre-purchase stage, with the accessibility of smartphones giving them the aura of time- and energy-saving devices (Boardman and McCormick 2018; Kim et al., 2019; Singh and Swait 2017). The need for comfort – that is, the social and emotional need for personal contacts to reduce perceived risks – correlates with the use of fewer channels along the customer journey and with the choice of brick-and-mortar stores in particular (Boardman and McCormick 2018; Schröder and Zaharia 2008). This accords with the common perception of offline channels as personal in character (Albesa 2007; Barwitz and Maas 2018; Hu and Tracogna 2020), but may change with the continuous rise of social networks, social media, and other channels providing for virtual human contact (Dalla Pozza 2014; Fiestas and Tuzovic 2021). The need for touch, likewise typically related to offline channels (Acquila-Natale and Iglesias-Pradas 2021; Cho and Workman 2011; Konuş et al. 2008), may drive webrooming, with customers searching for information online, but visiting a store to assess a product’s quality and make any purchase that arises from the process (Lee and Jung 2020; Maggioni et al. 2020). Channel switching may also occur due to cost avoidance motives, which appear to lead customers to less personal and less interactive channels such as the internet (Barwitz and Maas 2018; Boardman and McCormick 2018), notwithstanding the finding by Kukar-Kinney and Close (2010) that waiting for sale or lower prices is one of the main reasons for online shopping cart abandonment and subsequent purchase in brick-and-mortar stores. For redress-seeking, a need unique to the after-sales stage, Miquel-Romero et al. (2020), Mattila and Wirtz (2004) and Frasquet et al. (2021) showed that customers tend to visit interactive complaint channels such as brick-and-mortar stores. If dissatisfaction with a product is high, the importance of a channel’s convenience wanes and customers focus more on redress – a scenario that exemplifies the influence of customer needs on the relative significance of perceived channel characteristics. Where a customer seeks only to “vent”, by contrast, they tend to choose remote channels such as email for complaints due to a desire for a convenient outlet for frustration and unhappiness that avoids confrontation with in-store staff (Mattila and Wirtz 2004).

#### Situational and contextual factors

In some situations, customers are not in a position to choose every channel they may ideally want (Chocarro et al. 2013). Customers who generally prefer online channels may visit a store if they are experiencing poor internet connectivity or do not have their smartphone to hand. Those with offline preferences, meanwhile, may use the internet if they need an immediate solution to a task, such as the opportunity to avoid standing in line to buy a ticket for an imminently departing train (Cheng and Huang 2014). Other customers may discount some channels from the outset due to not having the required facilities, such as credit cards for online shopping (Lu and Rucker 2006). Channel choice and cross-channel switching along the customer journey may therefore not always constitute intentional or planned behavior (Maggioni et al. 2020). The proven influence of marketing campaigns and a customer’s social setting on channel choice is indicative of its potential dependence on context (Bilgicer et al. 2015). Situational and contextual factors, including time, place, communication and channel availability (see Table 5), impact the opportunity and ability to use various channels, influencing customers’ choice of channel both directly and indirectly.

**Table 5:**
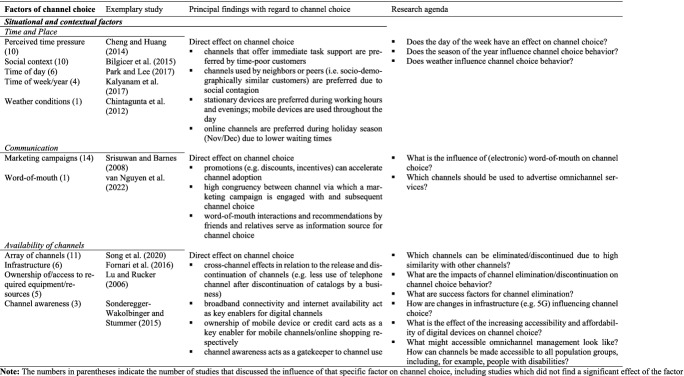
Factors of channel choice investigated in the articles reviewed – 3. Situational and contextual factors

One situational factor identified in the channel choice literature is *time*, for instance with regard to the time of day, week or year. Park and Lee (2017) found use of mobile channels throughout the day due to their ubiquity and portability, but a concentration of desktop device use between typical working hours of 9 a.m. and 6 p.m. (Park and Lee 2017). Time of day also has a direct impact on access to many brick-and-mortar stores due to opening hours (Chocarro et al. 2013). Other work has noted a preference for the internet over other channels during the months of November and December (Bilgicer et al. 2015; Kalyanam et al. 2017), which Kalyanam et al. (2017) attribute to the advantages of the online channel (no standing in line and no waiting time) during the holiday shopping season. A further dimension of time’s impact on channel choice relates to the requirements and motivations of customers who have little time available; these consumers will focus on the speed of the process they can achieve by using a particular channel (Cheng and Huang 2014; Oppewal et al. 2013) and place greater emphasis on utilitarian needs such as convenience-seeking (Barwitz and Maas 2018). Unsurprisingly, a lack of time limits the number of channels utilized along the customer journey (Oppewal et al. 2013) and seems to promote the use of mobile devices (Wagner et al. 2020).

Regarding *place* and social surrounding, it is shown that customers tend to adopt the same channels as their neighbors (local contagion) and as socio-demographically similar customers (homophily) (Bilgicer et al. 2015). This effect, however, diminishes over time and is consequently particularly strong among new customers (Bilgicer et al. 2015).^4^ Physical distance between a customer’s location and offline channels manifests to the customer as a transaction or access cost (Soysal and Krishnamurthi 2016), while proximity to brick-and-mortar stores will prompt a customer to use them (Bilgicer et al. 2015; Soysal and Krishnamurthi 2016). Location may also impact the use of online channels due to regionally poor internet coverage or broadband connectivity (Fornari et al. 2016).

*Communication*, especially in the form of corporate marketing campaigns, is an important explanatory factor in channel choice behavior (Bilgicer et al. 2015). Promotion of a specific channel via discounts, incentives, or similar may boost positive attitudes toward the channel’s use (Srisuwan and Barnes 2008) and accelerate channel adoption (Bilgicer et al. 2015; Sun et al., 2019; Venkatesan et al. 2007). Many studies indicate congruency between marketing campaigns and channel choice; a campaign conducted via emails, for instance, may engender increased customer willingness to choose an online channel as they already use a digital device to access the mail (Ansari et al. 2008; Bilgicer et al. 2015; Kalyanam et al. 2017; Mark et al. 2019; Polo and Sese 2016).

Finally, and evidently, the *availability of channels* directly influences channel choice. Channel awareness refers to the customer’s knowledge of channels’ existence and those within her consideration set. The availability of infrastructure and ownership of required equipment, such as a PC, smartphone, and/or credit card, act as key enablers for most digital channels (Boulay et al. 2014; Lu and Rucker 2006; Madden et al. 2017).

#### Customer characteristics

Customer characteristics have an indirect influence on channel choice (see Table 6). In terms of the frequently studied variable of customer age, younger customers are more likely to use online channels along the customer journey than their older counterparts (see, for example, Brand et al. 2020; Keyser et al. 2015), and the pattern re-emerges in relation to mobile and other relatively innovative channels such as social media (examples are in Dorie and Loranger 2020; Singh and Jang 2022; Singh and Swait 2017). However, no customer chooses digital channels just because she is young. Instead, younger generations that have grown up with digital technologies have greater experience and confidence in their use (Lipowski and Bondos 2018); they also tend to perceive fewer risks in online channels (Li et al., 2019). As a group, younger customers are more price-conscious and place less emphasis on privacy issues (Madden et al. 2017), which factors further reduce their focus on perceived online channel risks and create emphasis on needs such as cost avoidance. Very young customers, however – children aged 6 to 12 years – may view online channels critically and prefer offline channels for shopping (Boulay et al. 2014); this may be due to the greater importance of other needs, such as enjoyment-seeking, among this group, or to varying perceptions of channel attributes (for example, children may find physical stores easier to navigate than online channels), or to situational aspects which inhibit the use of certain channels, such as a lack of access to online payment options. Similarly, Alt et al. (2021) found an inverse U-shaped relationship between age and choice of digital channels for very complex products such as life insurance. This can be explained by the lack of financial literacy and the resulting need for information among the youngest insurance customers and the lack of experience with digital channels and the resulting perception of channel risks by the oldest customers (Alt et al. 2021). These findings lend further support to the argument that customer characteristics influence channel choice only indirectly due to the diversity of needs, perceptions, situations, and contexts that characterize the approach of individual customers to a channel.

**Table 6:**
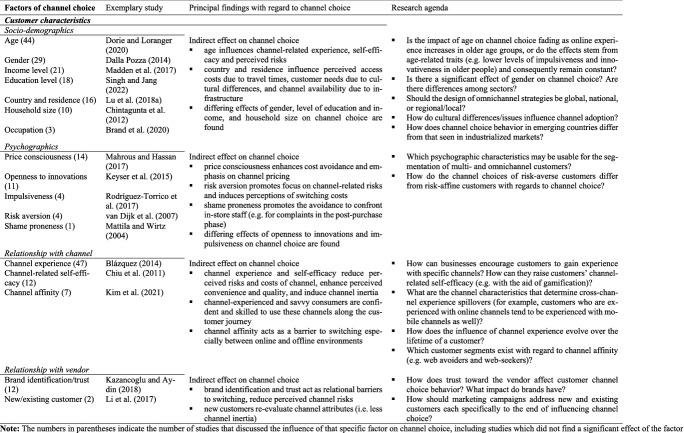
Factors of channel choice investigated in the articles reviewed – 4. Customer characteristics

Beside customer age, *socio-demographic factors* identified in the channel choice literature include gender, income and education level, country and residence, household size and occupation. A customer’s country and residence influences channel choice in several ways, including the potential effect of cultural dimensions on customer needs and consequently on channel selection (Ali et al. 2021; Hofstede et al. 2010; Lu et al. 2018a; Park and Kim 2018), physical distance from offline channels (Lim et al. 2021; Sousa et al. 2015; Soysal and Krishnamurthi 2016), and availability or otherwise of channels due to infrastructural conditions such as internet coverage or broadband connectivity (Fornari et al. 2016).

*Psychographic factors* such as customer price consciousness, openness to innovation, impulsiveness, and risk aversion serve in numerous studies to segment various types of multichannel and omnichannel customers (see, for example, Brand et al. 2020; Konuş et al. 2008; Maggioni et al. 2020; Sands et al. 2016; Sebald and Jacob 2020). Hallikainen et al. (2019), for instance, demonstrated that “digital channel enthusiasts” show high levels of innovativeness and low levels of technology-related insecurities. Keyser et al. (2015), by contrast, found innovativeness not to be a significant covariate for the segmentation of multichannel customers. Similarly, the literature points to various differing effects of price consciousness, impulsiveness, and risk aversion on channel choice.

Consumers also diverge with respect to their *relationship with the channel and with the vendor*, both of which evolve and change over the customer’s lifetime and play an important role in channel choice. The level of customers’ experience with a channel (“channel experience”) receives the most mentions in the literature as a factor in channel choice, occurring in almost 40% of the articles reviewed (n = 47). The extent of existing channel experience lessens emphasis on the channel’s perceived disadvantages, such as perceived risks (Xu and Jackson 2019b) and perceived access, transaction and search costs (Kalyanam et al. 2017; Konuş et al. 2014), as well as enhancing its perceived benefits, which might include convenience (Blázquez 2014) and media richness (Lipowski and Bondos 2018), and boosting customers’ confidence and sense of self-efficacy in their use of the channel (Chiu et al. 2011; van Dijk et al. 2007). Channel experience, then, can act as a facilitator or a barrier, particularly with regard to digital channels (Eckl and Lingenfelder 2021; Hallikainen et al. 2019; Sousa et al. 2015). Generally speaking, customers tend to use the channels with which they are experienced (Albesa 2007; Hu and Tracogna 2020; Polo and Sese 2016). The level of channel experience a customer has can thus explain channel inertia (i.e. use of the same channel throughout the customer journey and lifetime) and the slow adoption of digital channels in relation to some sectors (Frambach et al. 2007; Sousa and Voss 2012). For instance, Filotto et al. (2021) found that a lack of online experience among customers may be the most critical barrier to the adoption of internet banking. All this notwithstanding, in certain situations which may represent disruptions of the shopping process, such as the switching of retailers, customers may re-evaluate the benefits and costs of channels, and renounce previous habits (Li et al. 2017).

#### Characteristics of the product or service in question

Another category with indirect influence on channel choice is the *characteristics of the product or service* of interest (see Table 7). Customer needs, and the importance of perceived channel characteristics, vary in accordance with the pricing, involvement, risk level, and complexity of the specific product or service in question (Guo et al. 2021; Kondo and Okubo 2022). Like customer characteristics, then, the type of product or service affects channel choice indirectly. Expensive products, for instance, motivate a higher need for information and risk reduction (Kakalejcík et al. 2019; Xu and Jackson 2019a). The influence of involvement (Chocarro et al. 2013), risk level (Heitz-Spahn 2013), and complexity (Keyser et al. 2015; Kim et al. 2019), each factors closely related to price, is similar. Customers interested in purchasing high-priced, risky or complex types of products or services, may frequently avoid channels associated with an inherent perception of risk, such as the mobile channel (Park and Lee 2017; Sun et al. 2019). Such products may also be associated with showrooming as customers try to satisfy their need for information and service by visiting brick-and mortar stores at the pre-purchase stage, but switch to online channels for the actual purchase (Daunt and Harris 2017; Guo et al. 2021; van Nguyen et al. 2022).

**Table 7:**
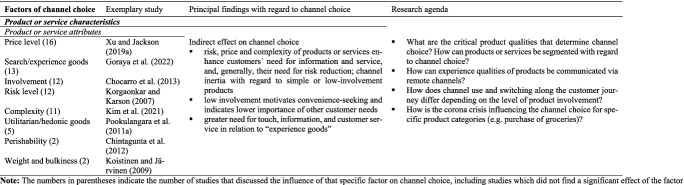
Factors of channel choice investigated in the articles reviewed – 5. Product or service characteristics

The literature on channel choice makes two sets of distinctions regarding types of products and services: they may be either “search” or “experience” goods (see, for example, Goraya et al. 2022), and additionally, along lines similar to those identifying divergent customer needs, either utilitarian or hedonic goods (cf. Eckl and Lingenfelder 2021; Singh and Swait 2017). Researchers (Lee and Jung 2020; Park and Lee 2017; Verhagen et al. 2019) find relatively greater needs for touch, information, and customer service in relation to experience goods, which are those containing significant proportions of qualities that elude determination prior to their use and only emerge in the course of that use (Nelson 1970). Search goods – products whose major qualities are more amenable to ascertainment prior to use – appear to evoke fewer needs of this type. This said, the literature shows no clear effect of these qualities on channel choice, variously reporting a tendency for experience goods to be purchased online (Park and Lee 2017) and to cause webrooming (purchase offline; Lee and Jung 2020), while other authors note no significant trends in this respect (Chocarro et al. 2013; Voorveld et al. 2016). Similarly, no significant difference emerges between channel choices made for utilitarian and hedonic product purchases (Pookulangara et al. 2011a; Singh and Swait 2017). Other product categories may be linked to customers´ channel choice. Chintagunta et al. (2012), for instance, found more frequent use of online channels for heavy or bulky items due to convenience-seeking and a tendency to visit brick-and-mortar stores for perishable goods, probably due to a high need for touch and necessity of immediate possession.

#### Stage of customer journey

Several studies (e.g. Ali et al. 2021; Barwitz and Maas 2018; Frambach et al. 2007; Frasquet et al. 2015; Gensler et al. 2012) note strong variations in customer needs, and consequently in the importance of perceived channel characteristics, by the stage of the customer journey in question. High needs for information at the pre-purchase stage give way to an emphasis on cost and risk avoidance when it comes to purchase (Kollmann et al. 2012; Polo and Sese 2016; van Dijk et al. 2007), and factors such as redress-seeking may gain ascendance in the after-sales phase (Miquel-Romero et al. 2020). These variations may motivate differences in channel choice. The types of channel switching along the customer journey which receive most attention in the articles reviewed are webrooming (cf., for example, Kim et al., 2019) and showrooming (e.g. Schneider and Zielke 2020). Webrooming appears more prevalent (Frasquet et al. 2015; Guo et al. 2021; Schröder and Zaharia 2008; Zhai et al. 2017), in line with the typical characteristics of each channel; the pre-purchase stage may feature use of online channels for convenient and easy information-gathering, and a later switch to offline channels for making the purchase may be motivated by perceived risk reduction and a desire to make use of the sensory and customer care benefits of brick-and-mortar stores (Herrero-Crespo et al. 2022). However, channel-switching along the customer journey is not the typical case, especially in relation to non-complex and non-expensive types of product, with several studies finding use of the same channel for information-gathering and the subsequent purchase (examples are Cao 2012; Gensler et al. 2012; Noble et al. 2005; Oppewal et al. 2013). Even in an omnichannel environment, customers tend to be either online- or offline-focused throughout the pre-purchase and purchase stages (Acquila-Natale and Iglesias-Pradas 2021; Valentini et al. 2020). This inertia appears to fall away at the after-sales stage (Frasquet et al. 2019; Keyser et al. 2015; Miquel-Romero et al. 2020), perhaps due to a reduced importance of channel convenience and a greater emphasis on other channel attributes such as personal contact and interactivity in this phase (Miquel-Romero et al. 2020). This would also explain the frequent choice of physical stores and social media channels for complaints (Dalla Pozza 2014; Frasquet et al. 2019; Miquel-Romero et al. 2020). Interestingly, when customers choose a complaint channel for a second complaint after an unsatisfactory first attempt, they tend to switch channels again; probably to take advantage of synergy effects between the channels (Frasquet et al. 2021).

### An integrated framework of channel choice

**Fig. 3:**
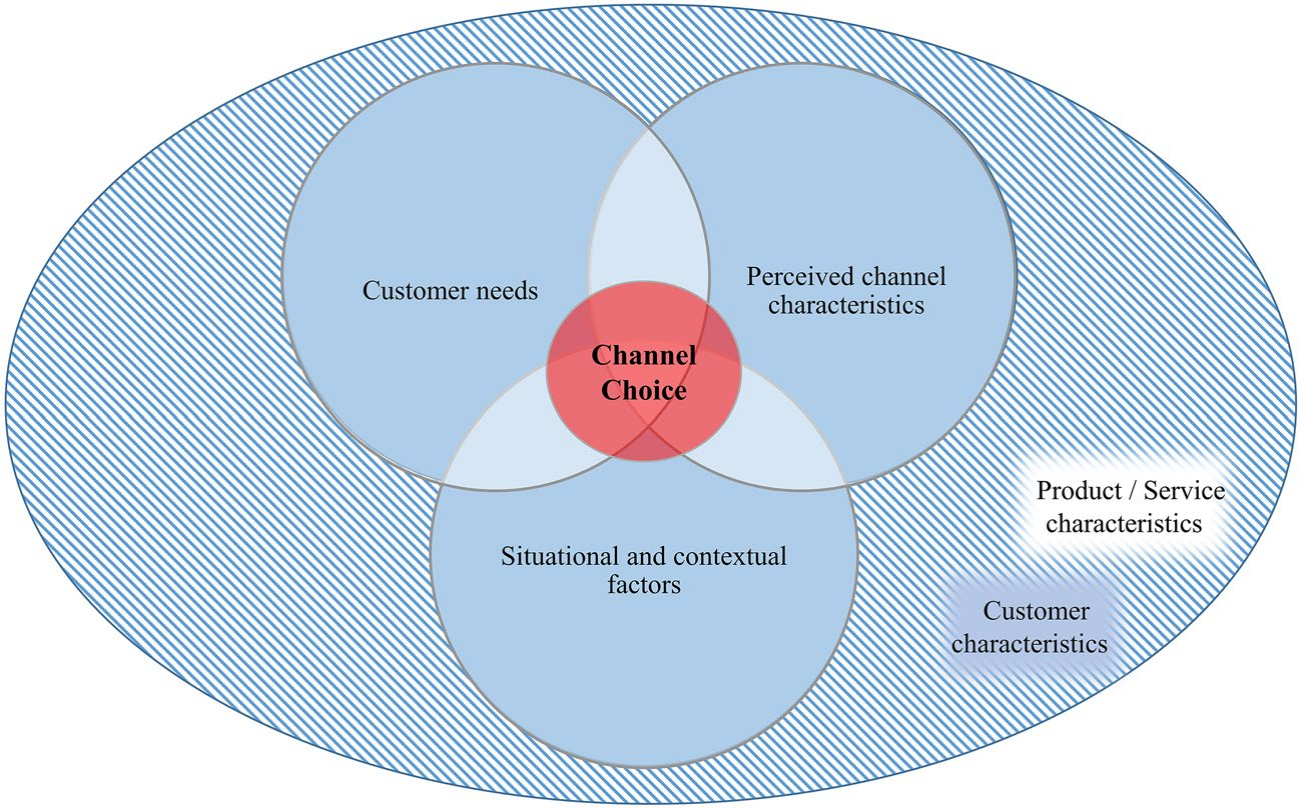
The conceptual framework

In an omnichannel environment, customers can choose the most efficient channel in accordance with its perceived utility in any situation (Gensler et al. 2012; Hosseini et al. 2018). The ultimate choice of channel depends on a number of directly and indirectly influential factors that often occur together and relate closely to and reciprocally impact one another; this calls for an integrated framework for the mapping of factors in channel choice (Gensler et al. 2012; Miquel-Romero et al. 2020). The conceptual framework we present here is fundamentally a Venn diagram illustrating that customers´ channel choice is directly influenced by an interplay of perceived channel characteristics, customer needs and situational or contextual factors, and indirectly influenced by customer characteristics and product or service characteristics (Fig. 3): Customers tend to choose channels whose characteristics give them the perceived ability to meet their needs (intersection between customer needs and perceived channel characteristics; Cervellon et al. 2015; Noble et al. 2005). However, there are situations and contexts that limit or inhibit the selection of channels – such as time pressure and a lack of required devices—or highlight new channels – an example might be marketing campaigns (Chocarro et al. 2013). Characteristics of customers and of products or services have a decisive influence on situational and contextual factors and on customers’ assessment of channel characteristics and of their needs.

## Suggestions for future research

The literature on channel choice is driven by the need to understand customer behavior in a constantly evolving channel environment. Its underlying objective is to derive implications for businesses that offer more than one channel. To this end, several studies segment customers, to provide paths to appropriate adaptation of channel management and marketing strategies (see, for example, Cheng and Huang 2014; Keyser et al. 2015; Lee and Jung 2020). Others investigate the adoption of digital and mobile channels by customers for the purpose of understanding cross-channel effects and switching of channels along the customer journey (e.g. Singh and Jang 2022; Yang et al. 2013). Research in this area now finds itself confronted with an ever-increasing array of possible channels and a proliferation of mobile devices that are driving the evolution of the multichannel environment into an omnichannel environment which remains under-researched in terms of the scene it sets for channel choice (Lemon and Verhoef 2016; Saghiri et al. 2017). Unanswered questions remain regarding factors of channel choice in this new environment and the management and marketing tactics arising therefrom (see Tables 3–7). Further, extant channel choice research focuses on a limited number of industries and countries, and its findings require validation via the introduction of other methods and research designs. In addition, the studies lack theoretical foundations and frameworks. Against this backdrop, this section discusses the research agenda for factors of channel choice and potential areas for new research in this field.

### Research agenda for factors of channel choice

*Channel characteristics:* We identified quality, convenience, risk, and cost as the principal characteristics of a channel in the channel choice literature. However, most studies investigate these characteristics separately (e.g. Arora et al. 2017; Oppewal et al. 2013), risking the omission or overlooking of possible interrelationships and spillover effects. A broader, more holistic view of channel characteristics would be desirable (Dennis et al. 2016; Rodríguez-Torrico et al. 2017; van Dijk et al. 2007). We are in need of further research classifying channels according to their similarities or dissimilarities in terms of characteristics; this would improve the effectiveness of channel management, enabling, for example, the discontinuation of channels that customers perceive as very similar to others and therefore as superfluous (Hosseini et al. 2018). Numerous studies highlight both positive (cross-channel synergies; see, for example, Fornari et al. 2016; Kumar et al. 2019; Verhagen and van Dolen 2009; Yang et al. 2014) and negative (cross-channel competition or cannibalization; e.g. Bilgicer et al. 2015; Li et al. 2017; Lim et al. 2021) cross-channel effects. A holistic understanding of these effects along the customer journey could be of significant benefit to channel management (Bilgicer et al. 2015; Dorie and Loranger 2020; Trenz et al. 2020; Yang et al. 2014).

Channel characteristics are constantly evolving alongside emerging new technologies and digital devices (Sands et al. 2016). Further research could, for example, examine the impact of virtual and augmented reality technologies such as virtual dressing rooms on perceptions of channel characteristics (Eckl and Lingenfelder 2021; Kim et al. 2021). We also note the changing role of some channels in the omnichannel environment; brick-and-mortar stores, for instance, are gaining in significance for the pre- and post-purchase stages, but may be taking on a more complementary role for the actual purchase (Fornari et al. 2016; Miquel-Romero et al. 2020).^5^ In light of these shifts, the question emerges as to how retailers might adapt their assortment, prices and communications to channel-specific characteristics and the channels’ new roles while implementing effective omnichannel management in the sense of providing a seamless and integrated multichannel experience (Blázquez 2014; Singh and Jang 2022; Valentini et al. 2020).

*Customer needs:* The optimization of customer experience is an integral part of omnichannel management (Verhoef et al. 2015). Multi- and omnichannel businesses require an understanding of both utilitarian and hedonic needs. Some traditional benefits of specific channels, such as human contact and immediate quality evaluation in physical stores, are becoming less clearly defined and less unambiguously attributable (Hallikainen et al. 2019); social media, for instance, provide interactivity and human contact and thus meet customers’ need for comfort (Dalla Pozza 2014). Future research should investigate the influence of emerging channels and technologies on the fulfillment of customer needs (Dalla Pozza 2014; Lee and Jung 2020), and specifically the influence of perceived benefits of the online channel (including vast ranges, customer-centeredness, ease of use) on customer needs in general, as perceptions of online channels’ benefits may conceivably spill over to customers’ needs and expectations when using other channels (Santos and Gonçalves 2019; Verhagen et al. 2019).

While utilitarian needs tend to have a greater influence on channel choice than hedonic needs, the investigation of the latter may gain significance as the proportion of product and service types offered through a variety of channels rises (Lee and Jung 2020). Future research should therefore seek to ascertain any differences in the impact of hedonic needs on channel choice among industries or countries (Cervellon et al. 2015; Koistinen and Järvinen 2009; Noble et al. 2005). In our time of climate crisis and heightened ecological consciousness, needs such as sustainability orientation are likely to acquire prominence and require corresponding attention from researchers (Cervellon et al. 2015).

*Situational or contextual factors:* Situational factors are difficult for businesses to influence, which makes it all the more important that they understand their potential impact on channel selection so they can adapt their channel management accordingly (Chocarro et al. 2013). One example in this regard might be the finding by Koistinen and Järvinen (2009) that customers preferred specific channels (such as marketplaces) for their grocery purchases on weekends due to their hedonic characteristics. Whether the day of the week or the season of the year influence channel choice in general, however, remains unclear (Bussière 2011). Future research could examine such potential differences more closely and generalize the findings for other sectors. A further notable research gap relates to communication, more specifically to the impact of (electronic) word of mouth on channel selection (Bilgicer et al. 2015; Kim et al. 2019). The ever-evolving infrastructure, accessibility, and affordability of digital devices may also be driving change in channel choice behavior (Frasquet et al. 2015; Singh and Swait 2017; Wagner et al. 2020). We encourage researchers to identify the sectors in which digital devices complement established channels along the customer journey and those in which the former act more as substitutes for the latter (Singh and Swait 2017; Voorveld et al. 2016). The current COVID-19 pandemic and the associated measures have exercised a major influence on channel choice behavior and served as an object lesson in the power of situational factors; social distancing and lockdowns, for instance, push up the access costs of brick-and-mortar stores (Wang et al. 2021a). There is clearly much research to be done into the pandemic’s short- and long-term impact on channel choice (Filotto et al. 2021; Frasquet et al. 2021; Kondo and Okubo 2022).

*Customer characteristics:* Socio-demographic and psychographic characteristics of customers find entry into most studies on channel choice, as either independent or control variables. However, the studies do not speak with one voice on the impact of gender, education level, income level, household size, openness to innovation, and impulsiveness on channel choice. It would be of benefit to the field for future research to empirically identify any sectors within which these factors have a significant influence on channel choice and may therefore help inform customer segmentation (Boardman and McCormick 2018; Dalla Pozza et al. 2018; Sebald and Jacob 2020). In terms of customer location, the question remains as to whether global design or local adaptation of channel strategies is preferable in the light of differences in customer needs (Korgaonkar and Karson 2007; Lu et al. 2018a; Sebald and Jacob 2020).

Customer characteristics also involve the customer’s relationship with channels and with the vendor. We perceive a need for research into ways to enhance the extent of customers´ experience with a channel as one of the major factors of channel choice (Hu and Tracogna 2020; Xu and Jackson 2019b) and for analysis of cross-channel experience spillovers (Gensler et al. 2012). While some studies investigated how channel choice evolves over time (see, for example, Valentini et al. 2011), we are as yet without an updated and omnichannel perspective (Hu and Tracogna 2020). Additionally, with regard to relationship with the vendor, the role of brands in channel choice behavior remains unclear despite its potential importance (Boardman and McCormick 2018; Cervellon et al. 2015; Frasquet et al. 2015; Korgaonkar and Karson 2007). Practitioners would further benefit from studies on the differential impact of marketing campaigns on new or existing customers in terms of their susceptibility to persuasion to adopt a particular channel (Valentini et al. 2011).

*Product or service characteristics:* The distinctions between types of product or service that are examined in some work on channel choice (i.e. “search” versus “experience” goods, utilitarian versus hedonic goods) do not result in clear evidence. The result is a need for future research to examine possibilities for higher-quality segmentation criteria with regard to channel choice for a range of types of product and service (Chang et al. 2017; Frasquet et al. 2015; Gensler et al. 2012; Heitz-Spahn 2013; Kondo and Okubo 2022; Pookulangara et al. 2011a); such work has the potential to inform businesses’ omnichannel management. One criterion for segmentation might be the level of customer´s involvement with the product (Arora and Sahney 2018; Gallant and Arcand 2017; Konuş et al. 2008), although the number of studies investigating the impact of involvement level on channel choice is low (they include Chocarro et al. 2013; Frasquet et al. 2015; Voorveld et al. 2016).

### Research agenda for theories, contexts, and methods

This section is based on the TCM (i.e. theories, contexts, and methodology) framework introduced by Paul et al. (2017). It first details the research agenda for omnichannel management with regard to theories, before proceeding to examine current gaps and needs in relation to two fundamental contexts of research in the field, location (country) and business sectors. Finally, it notes potentially promising methodologies in this context.

In line with Mishra et al. (2021), we find that the multi- and omnichannel literature lacks theoretical foundations and models. Only a small number of articles on customers´ channel choice specifically indicate the application of theories (e.g., Pookulangara et al. 2011a, 2011b). However, these theories often fail to provide a holistic picture of customers´ channel choice. For instance, multi-attribute utility theory delivers theoretical foundations for the investigation of channel choice behavior, suggesting that customers choose channels along their customer journey in accordance with the utility they expect from them. However, this theory does not account for other important determinants of channel choice, such as situation, context, and customer characteristics, and consequently requires modification (Maggioni et al. 2020). We would encourage future research in this area to extend such established theories or develop new models and frameworks for an improved theoretical understanding of customers´ channel choice. Another area within which future research might broaden its horizons relates to the channels the studies consider. Despite the variety of channels in existence, a significant proportion of the studies reviewed (n = 47) persists in distinguishing only between online and brick-and-mortar formats (see, for example, Bilgicer et al. 2015; Kollmann et al. 2012; Wang et al. 2016). The fact that only 22 studies (about 17% of all articles) focus exclusively on omnichannel management accentuates the necessity of extending the research vista on channel choice. Some studies call for the inclusion of social media and social networks in this work (Lee and Jung 2020; Miquel-Romero et al. 2020; Polo and Sese 2016; Singh and Swait 2017).

The major portion of the articles reviewed is from the U.S. (n = 34), China (n = 13), and Western Europe (U.K.: n = 10; Spain = 10; Germany: n = 9). The complete absence of studies from Africa or South America (apart from three studies from Egypt; Ali et al. 2021; Hussein and Kais 2021; Mahrous and Hassan 2017) stands in sharp contrast to these figures. Research in emerging countries and comparison with developed markets would appear to constitute a pressing need in view of the rising prevalence of new technologies and channels in these areas (Dalla Pozza et al. 2018; Mahrous and Hassan 2017). Several studies wish to see cross-cultural and transnational research done in this area (e.g. Frasquet et al. 2015; Mattila and Wirtz 2004; Sebald and Jacob 2020). Although there are some cross-industry studies on channel choice (examples are Kim et al. 2019; Verhagen et al. 2019), a holistic comparative study across various industrial and service sectors has yet to take place (Barwitz and Maas 2018; Jerath et al. 2015). The current literature largely excludes some types of product, such as cars and other vehicles. Many studies highlight the need for validation of their findings in other sectors and for other product categories in order to ascertain their generalizability (e.g. Chiou et al. 2017; Lee and Kim 2009; Santos and Gonçalves 2019). A particular gap appears to relate to research on experience goods (Cao 2012; Kim et al. 2021; Trenz et al. 2020). Additionally, future research is encouraged to examine the whole customer journey to expand the extensive literature on channel switching between the pre-purchase and the purchase stage (Eckl and Lingenfelder 2021; Hussein and Kais 2021).

In terms of methodology, most studies in our review are quantitative (n = 110), primarily using surveys; a number of articles (n = 25) call for the validation of their findings via longitudinal studies and an extended data collection period (see, for example, Hu and Tracogna 2020; Sun et al. 2020). A long-term examination of customers’ channel use would promote a holistic understanding of evolving patterns along the customer journey and support the analysis of trends (Frasquet et al. 2015; Gensler et al. 2012). Several studies (such as Jerath et al. 2015; Kukar-Kinney and Close 2010) highlight the potential of new data bases such as clickstream data and transcripts of telephone conversations. Experimentally-based methods or mixed methods designs may assist in the exploration and augment the understanding of causal relationships around channel choice behavior; mixed-methods approaches in particular appear to have considerable capacity to uncover the customer perspective in an omnichannel environment and validate previous findings (Shen et al. 2018). Replicating existing studies in additional contextual environments and with different or extended samples is highly recommended (Block and Kuckertz 2018; Jebarajakirthy et al. 2021; Wang et al. 2013).

## Conclusion

The present study conducted a systematic literature review to the end of identifying the factors involved in channel choice which appear in the scientific literature on this topic over the last two decades. The review sought to enhance our understanding of customer behavior in multi- and omnichannel environments – a vital factor in successful omnichannel and customer experience management (Verhoef et al. 2015). For the review, we retrieved 128 papers from three bibliographic databases, namely EBSCO Host, Scopus and Web of Science, and carried out descriptive and qualitative analysis on them using an inductive coding approach. The descriptive analysis indicates that the recent move from multi- to omnichannel environment has given notable impetus to customer channel choice research; over 60% of the papers on channel choice we examined in this review were published in the last six years (to May 2022) and this body of work continues to grow at a rapid rate. What remains missing, in view of the limitation of most studies to specific contexts (such as industries, countries, considered channels or customer journey phases), is an integrated understanding of the customer perspective on channel choice. This SLR, in attempting to synthesize and aggregate the current literature on channel choice, has uncovered 66 different factors of customers´ channel choice, each assignable to five broader categories. It shows that an interplay of perceived channel characteristics, customer needs, and situational or contextual factors influences chancel choice behaviors directly, and customer characteristics and product or service characteristics influence them indirectly. Alongside its presentation of an integrated conceptual framework comprising these relationships, this study has detailed a comprehensive agenda for future research into the omnichannel environment with regard to theories, contexts, and methods, with a particular emphasis on factors of customers´ channel choice.

The integrated conceptual framework of customers´ channel choice proposed in this review and our recommendations for potential future research contribute to the literature in this field by advancing knowledge around customer behavior for academia and practitioners, providing a systematic overview of the impact of various factors on channel choice, and precisely formulating over 50 unanswered questions for research going forward (see Tables 3, 4, 5, 6 and 7). For managers, the findings of the review give important insights into customers´ channel choice behaviors, occasioning opportunities for the improvement of customer experience and omnichannel management. First, these findings indicate that channel choice is a highly complex process to which a variety of different factors are material. If managers wish to influence channel choice or switching behavior, they have to acknowledge this multidimensional nature of the phenomenon. For instance, simply improving characteristics of a channel, such as service quality and ease of use, may not result in increased use of that channel by customers or in switching, due to the influence of other significant factors such as customer needs or situational circumstances. Second, our study suggests that the influence of customer and product or service characteristics on channel choice is indirect only. This would imply that managers seeking to improve channel management procedures should place greater weight on understanding the factors that are directly influential in channel choice. For customer segmentation on the basis of channel choice, for instance, managers will require a comprehensive understanding of differences in customer needs if they are to tailor channel strategies in accordance with these needs and improve customer experience. Several studies on channel choice strongly advise companies against eliminating traditional brick-and-mortar channels in order to avoid alienating customer segments with a high need for touch or need for comfort and to take account of product types that cause customers to emphasize specific channel characteristics such as staff support and immediate product availability (examples of such studies are, Acquila-Natale and Iglesias-Pradas 2021; Miquel-Romero et al. 2020; Singh and Jang 2022). Finally, while omnichannel management includes the synergetic management of channels and touchpoints, the heterogeneous findings of the studies included in this review suggest that there is no such thing as a one-size-fits-all channel strategy. The differing characteristics of the various channels address distinct customer needs and point to differences in suitability for various situations and contexts. For instance, mobile channels (i.e. smartphones and tablets) often find use for pre-purchase tasks, while the traditional online channel (i.e. stationary devices) may serve to make the actual purchase due to the availability of a larger screen size and fewer security concerns. As a consequence, managers should facilitate easy device switching along the customer journey and adapt their online content to various sizes of screen and other device characteristics (Haan et al. 2018).

This review is the first study to date to provide a systematic overview of the currently existing body of knowledge on customers´ channel choice; more generally, it is one of the first SLRs on multi- and omnichannel-related topics to center the customer´s point of view. Notwithstanding this contribution to the research landscape, the review is subject to some limitations. First, we had to restrict the search strings to specific terms such as “channel,” although articles falling within our criteria may have used other denotations (an example might be “interaction choice” (Barwitz and Maas 2018)), meaning we potentially missed some relevant work. This said, a SLR does not seek to identify every single article that has been published on a topic, but rather to create a relatively – as opposed to absolutely – complete census of relevant literature for the acquisition of a comprehensive, multi-dimensional perspective on the issue at hand (Webster and Watson 2002). Further, the partially subjective character of the screening process limits the reproducibility of the final body of articles to be included in the review. To increase the validity and reliability of the selection process, three researchers independently evaluated the studies´ eligibility. A further limitation appears in our finding that the current omnichannel literature still lacks a clear, concise and homogeneous definition of the term “channel”, which we would need if we are to aim at a consistent understanding of the concept (Wagner et al. 2020). We further note that, the processes of identifying determining factors of channel choice within the literature and assigning the factors to (sub-)categories were highly subjective due to the inductive method utilized for coding. Meta-analyses could advance the statistical assessment of quantitative studies on channel choice and thus validate the findings of this review (Paul and Criado 2020). A final limitation we wish to mention is located in the future; the constant and rapid evolution of multi- and omnichannel management means that factors of channel choice are susceptible to change, creating a possible need for replication of the review at some point yet to be defined.

## Data Availability

The datasets generated during and/or analyzed during the current study are available from the corresponding author upon request.

## Appendices

### Appendix A

See Table 8.

**Table 8:** Comparison of recent systematic literature reviews within the multi- and omnichannel environment with the present study

Author(s)	Title	Objective	Perspective	Category	Type of review^a^	Methodology^b^	Sample	Time span	Databases
Cai and Lo (2020)	Omni-channel management in the new retailing era: A systematic review and future research agenda	Identify general research domains within omnichannel management academia, illustrate evolvement for each domain and derive research agenda	Management	Omnichannel	Bibliometric	Bibliographic modeling (main path analysis), topic modeling (citation network cluster analysis)	192 articles	n.a.-2019 (March)	Web of Science
Gao et al. (2020)	Multichannel integration along the customer journey: a systematic review and research agenda	Systemize theoretical and empirical findings regarding multichannel marketing mix integration and influences on customer experience along the customer journey	Management	Multichannel	Review aiming for theory development	Descriptive content & thematic analysis	75 articles	1998–2018	Scopus, Web of Science
Gerea et al. (2021)	Omnichannel Customer Experience and Management: An Integrative Review and Research Agenda	Summarize and synthesize research regarding omnichannel customer experience and its management	Management	Omnichannel	Structured	Descriptive content & thematic analysis	50 articles	n.a	ACM Digital Library, IEEE Xplore, ProQuest, ScienceDirect, Scopus, SpringerLink, Web of Science
Hossain et al. (2019)	Multichannel integration quality: A systematic review and agenda for future research	Explore definitions, dimensions, and challenges of multichannel integration quality in services marketing	Management	Multichannel	Hybrid	Thematic analysis + qualitative analysis (in-depth interviews & focus groups discussions)	90 articles	2003–2018	ProQuest *[ABI/Inform]*, EBSCO Host *[Business Source Complete, Open Access Journals]*, ScienceDirect
Lopes et al. (2021)	A citation and co-citation bibliometric analysis of omnichannel marketing research	Elaborate a core knowledge for omnichannel marketing research	Management	Omnichannel	Bibliometric	Bibliographic modeling (citation analysis and co-citation factor analysis)	59 articles	1900–2019 (Oct.)	Web of Science *[Core collection]*
Mishra et al. (2021)	Consumer decision-making in omnichannel retailing: Literature review and future research agenda	Categorize and analyze key findings regarding consumer behavior and decision-making within omnichannel retailing	Customer	Omnichannel	Structured	Descriptive content & thematic analysis	131 articles	2011–2020 (April)	Web of Science
Salvietti et al. (2021)	An Overview on omnichannel research: Intellectual foundations and implications for research	Map the omnichannel research landscape and identify main research streams, theoretical foundations and future research agenda	Management	Omnichannel	Bibliometric	Bibliographic & topic modeling (co-citation cluster analysis)	314 articles	1985–2020	Web of Science *[Core collection]*
Wang et al. (2021b)	Satisfying consumers all around: a multidisciplinary view of omnichannel retail	Review current studies on omnichannel retail in information systems, operations and marketing research	Management	Omnichannel	Narrative type	Thematic analysis (narrative conceptualization)	33 articles	2014–2020 (June)	n.a
**This paper**	Factors of Channel Choice from a Customers´ Perspective in an Omnichannel Environment: A Systematic Literature Review	Summarize and analyze determining factors of channel choice in multi- and omnichannel retailing	Customer	Multi- and Omnichannel	Structured	Descriptive content & thematic analysis	128 articles	2000–2022 (May)	EBSCO Host *[Business Source Complete]*, Scopus, Web of Science *[Core Collection]*

^a^ According to Paul and Criado 2020; ^b^ According to Paul et al. 2021

### Appendix B

See Table 9.

**Table 9:** Final selection of articles on channel choice

Author (Year)	Title	Journal
Acquila-Natale and Iglesias-Pradas (2021)	A matter of value? Predicting channel preference and multichannel behaviors in retail	Technological Forecasting and Social Change
Albesa (2007)	Interaction channel choice in a multichannel environment, an empirical study	International Journal of Bank Marketing
Ali et al. (2021)	Investigating the Situated Culture of Multi-Channel Customer Management: A Case Study in Egypt	Journal of Global Information Management
Alt et al. (2021)	Digital touchpoints and multichannel segmentation approach in the life insurance industry	International Journal of Retail and Distribution Management
Ansari et al. (2008)	Customer Channel Migration	Journal of Marketing Research
Arora et al. (2017)^a^	Understanding consumer’s showrooming behaviour	Asia Pacific Journal of Marketing and Logistics
Barwitz and Maas (2018)	Understanding the Omnichannel Customer Journey: Determinants of Interaction Choice	Journal of Interactive Marketing
Becker et al. (2017)	Cross-Industrial User Channel Preferences on the Path to Online Purchase: Homogeneous, Heterogeneous, or Mixed?	Journal of Advertising
Bezes (2016)^a^	Comparing online and in-store risks in multichannel shopping	International Journal of Retail and Distribution Management
Bilgicer et al. (2015)	Social Contagion and Customer Adoption of New Sales Channels	Journal of Retailing
Blázquez (2014)	Fashion Shopping in Multichannel Retail: The Role of Technology in Enhancing the Customer Experience	International Journal of Electronic Commerce
Boardman and McCormick (2018)	Shopping channel preference and usage motivations: Exploring differences amongst a 50-year age span	Journal of Fashion Marketing and Management
Boulay et al. (2014)	When children express their preferences regarding sales channels: Online or offline or online and offline?	International Journal of Retail and Distribution Management
Brand et al. (2020)	‘Online Omnivores’ or ‘Willing but struggling’? Identifying online grocery shopping behavior segments using attitude theory	Journal of Retailing and Consumer Services
Cao (2012)^a^	The relationships between e-shopping and store shopping in the shopping process of search goods	Transportation Research Part A: Policy and Practice
Cervellon et al. (2015)	Shopping orientations as antecedents to channel choice in the French grocery multichannel landscape	Journal of Retailing and Consumer Services
Chang et al. (2017)	Applying push–pull-mooring to investigate channel switching behaviors: M-shopping self-efficacy and switching costs as moderators	Electronic Commerce Research and Applications
Cheng and Huang (2014)^a^	High speed rail passenger segmentation and ticketing channel preference	Transportation Research Part A: Policy and Practice
Chintagunta et al. (2012)^a^	Quantifying Transaction Costs in Online/Off-line Grocery Channel Choice	Marketing Science
Chiou et al. (2017)	Consumer choice of multichannel shopping: The effects of relationship investment and online store preference	Internet Research
Chiu et al. (2011)^a^	The challenge for multichannel services: Cross-channel free-riding behavior	Electronic Commerce Research and Applications
Cho and Workman (2011)	Gender, fashion innovativeness and opinion leadership, and need for touch: Effects on multi-channel choice and touch/non-touch preference in clothing shopping	Journal of Fashion Marketing and Management
Chocarro et al. (2013)^a^	Situational variables in online versus offline channel choice	Electronic Commerce Research and Applications
Choi and Park (2006)	Multichannel retailing in Korea: Effects of shopping orientations and information seeking patterns on channel choice behavior	International Journal of Retail and Distribution Management
Dalla Pozza (2014)	Multichannel management gets “social”	European Journal of Marketing
Dalla Pozza et al. (2018)	Multichannel segmentation in the after-sales stage in the insurance industry	International Journal of Bank Marketing
Daunt and Harris (2017)	Consumer showrooming: Value co-destruction	Journal of Retailing and Consumer Services
Dennis et al. (2016)	Does social exclusion influence multiple channel use? The interconnections with community, happiness, and well-being	Journal of Business Research
Dholakia et al. (2005)	Multichannel retailing: A case study of early experiences	Journal of Interactive Marketing
Dorie and Loranger (2020)	The multi-generation: Generational differences in channel activity	International Journal of Retail and Distribution Management
Eckl and Lingenfelder (2021)^a^	Determinants of Consumers’ Purchase Channel Preference in Omni-Channel Retailing	Marketing: ZFP – Journal of Research and Management
Eriksson and Nilsson (2007)	Determinants of the continued use of self-service technology: The case of Internet banking	Technovation
Falk et al. (2007)	Identifying cross-channel dissynergies for multichannel service providers	Journal of Service Research
Fiestas and Tuzovic (2021)	Mobile-assisted showroomers: Understanding their purchase journey and personalities	Journal of Retailing and Consumer Services
Filotto et al. (2021)	Shaping the digital transformation of the retail banking industry. Empirical evidence from Italy	European Management Journal
Fornari et al. (2016)	Adding store to web: migration and synergy effects in multi-channel retailing	International Journal of Retail and Distribution Management
Frambach et al. (2007)^a^	The impact of consumer Internet experience on channel preference and usage intentions across the different stages of the buying process	Journal of Interactive Marketing
Frasquet et al. (2015)	Identifying patterns in channel usage across the search, purchase and post-sales stages of shopping	Electronic Commerce Research and Applications
Frasquet et al. (2019)	Understanding complaint channel usage in multichannel retailing	Journal of Retailing and Consumer Services
Frasquet et al. (2021)	Complaint behaviour in multichannel retailing: a cross-stage approach	International Journal of Retail and Distribution Management
Gallant and Arcand (2017)^a^	Consumer characteristics as drivers of online information searches	Journal of Research in Interactive Marketing
Gensler et al. (2012)	Understanding consumers' multichannel choices across the different stages of the buying process	Marketing Letters
Goraya et al. (2022)	The impact of channel integration on consumers’ channel preferences: Do showrooming and webrooming behaviors matter?	Journal of Retailing and Consumer Services
Guo et al. (2021)	Webrooming or showrooming? The moderating effect of product attributes	Journal of Research in Interactive Marketing
Hallikainen et al. (2019)	Individual preferences of digital touchpoints: A latent class analysis	Journal of Retailing and Consumer Services
Harris et al. (2017)	Online and store patronage: a typology of grocery shoppers	International Journal of Retail and Distribution Management
Heitz-Spahn (2013)	Cross-channel free-riding consumer behavior in a multichannel environment: An investigation of shopping motives, sociodemographics and product categories	Journal of Retailing and Consumer Services
Herrero-Crespo et al. (2022)	Webrooming or showrooming, that is the question: explaining omnichannel behavioural intention through the technology acceptance model and exploratory behaviour	Journal of Fashion Marketing and Management
Hosseini et al. (2018)	Mindfully going omni-channel: An economic decision model for evaluating omni-channel strategies	Decision Support Systems
Hu and Tracogna (2020)	Multichannel customer journeys and their determinants: Evidence from motor insurance	Journal of Retailing and Consumer Services
Huang et al. (2017)^a^	Investigation of Chinese students' O2O shopping through multiple devices	Computers in Human Behavior
Hussein and Kais (2021)	Multichannel behaviour in the retail industry: evidence from an emerging market	International Journal of Logistics Research and Applications
Jebarajakirthy et al. (2021)	Deciphering in-store-online switching in multi-channel retailing context: Role of affective commitment to purchase situation	Journal of Retailing and Consumer Services
Jerath et al. (2015)	An Information Stock Model of Customer Behavior in Multichannel Customer Support Services	Manufacturing and Service Operations Management
Jo et al. (2021)	Who are the multichannel shoppers and how can retailers use them? Evidence from the French apparel industry	Asia Pacific Journal of Marketing and Logistics
Kakalejcík et al. (2019)^a^	Differences in Buyer Journey between High- and Low-Value Customers of E-Commerce Business	Journal of Theoretical and Applied Electronic Commerce Research
Kalyanam et al. (2017)	Basket Composition and Choice Among Direct Channels: A Latent State Model of Shopping Costs	Journal of Interactive Marketing
Kazancoglu and Aydin (2018)	An investigation of consumers’ purchase intentions towards omni-channel shopping: A qualitative exploratory study	International Journal of Retail and Distribution Management
Keyser et al. (2015)	Multichannel customer segmentation: Does the after-sales channel matter? A replication and extension	International Journal of Research in Marketing
Kim et al. (2019)	Understanding shopping routes of offline purchasers: selection of search-channels (online vs. offline) and search-platforms (mobile vs. PC) based on product types	Service Business
Kim et al. (2021)	Channel stickiness in the shopping journey for electronics: Evidence from China and South Korea	Journal of Business Research
Koistinen and Järvinen (2009)	Consumer observations on channel choices: Competitive strategies in Finnish grocery retailing	Journal of Retailing and Consumer Services
Kollmann et al. (2012)	Cannibalization or synergy? Consumers' channel selection in online-offline multichannel systems	Journal of Retailing and Consumer Services
Kondo and Okubo (2022)	Understanding multi-channel consumer behavior: A comparison between segmentations of multi-channel purchases by product category and overall products	Journal of Retailing and Consumer Services
Konuş et al. (2014)	The effect of search channel elimination on purchase incidence, order size and channel choice	International Journal of Research in Marketing
Konuş et al. (2008)	Multichannel Shopper Segments and Their Covariates	Journal of Retailing
Korgaonkar and Karson (2007)	The influence of perceived product risk on consumers' e-tailer shopping preference	Journal of Business and Psychology
Kukar-Kinney and Close (2010)	The determinants of consumers’ online shopping cart abandonment	Journal of the Academy of Marketing Science
Kumar et al. (2019)	Why Do Stores Drive Online Sales? Evidence of Underlying Mechanisms from a Multichannel Retailer	Information Systems Research
Lee and Kim (2009)	Gift shopping behavior in a multichannel retail environment: The role of personal purchase experiences	International Journal of Retail and Distribution Management
Lee and Jung (2020)	Fashion consumers’ channel-hopping profiles by psychographics and demographics	International Journal of Market Research
Li et al. (2019)	Modeling and Analysis of Ticketing Channel Choice for Intercity Bus Passengers: A Case Study in Beijing, China	Journal of Advanced Transportation
Li et al. (2017)	Customer Channel Migration and Firm Choice: The Effects of Cross-Channel Competition	International Journal of Electronic Commerce
Lim et al. (2021)	The impact of mobile app adoption on physical and online channels	Journal of Retailing
Lipowski and Bondos (2018)	The influence of perceived media richness of marketing channels on online channel usage: Intergenerational differences	Baltic Journal of Management
Lu et al. (2018a)	Cross-national variation in consumers’ retail channel selection in a multichannel environment: Evidence from Asia–Pacific countries	Journal of Business Research
Lu and Rucker (2006)	Apparel acquisition via single vs. multiple channels: College students' perspectives in the US and China	Journal of Retailing and Consumer Services
Madden et al. (2017)	E-commerce transactions, the installed base of credit cards, and the potential mobile E-commerce adoption	Applied Economics
Maggioni et al. (2020)	Consumer cross-channel behaviour: is it always planned?	International Journal of Retail and Distribution Management
Mahrous and Hassan (2017)	Achieving Superior Customer Experience: An Investigation of Multichannel Choices in the Travel and Tourism Industry of an Emerging Market	Journal of Travel Research
Mark et al. (2019)	Catalogue as a tool for reinforcing habits: Empirical evidence from a multichannel retailer	International Journal of Research in Marketing
Mattila and Wirtz (2004)^a^	Consumer complaining to firms: The determinants of channel choice	Journal of Services Marketing
Miquel-Romero et al. (2020)	The role of the store in managing postpurchase complaints for omnichannel shoppers	Journal of Business Research
Montoya-Weiss et al. (2003)^a^	Determinants of Online Channel Use and Overall Satisfaction With a Relational, Multichannel Service Provider	Journal of the Academy of Marketing Science
Noble et al. (2005)	Consumer derived utilitarian value and channel utilization in a multi-channel retail context	Journal of Business Research
Oppewal et al. (2013)	Experimental analysis of consumer channel-mix use	Journal of Business Research
Park and Kim (2018)	A new approach to segmenting multichannel shoppers in Korea and the U.S	Journal of Retailing and Consumer Services
Park and Lee (2017)^a^	An empirical study on consumer online shopping channel choice behavior in omni-channel environment	Telematics and Informatics
Polo and Sese (2016)	Does the Nature of the Interaction Matter? Understanding Customer Channel Choice for Purchases and Communications	Journal of Service Research
Pookulangara et al. (2011a)	Explaining consumers' channel-switching behavior using the theory of planned behavior	Journal of Retailing and Consumer Services
Pookulangara et al. (2011b)	Explaining multi-channel consumer's channel-migration intention using theory of reasoned action	International Journal of Retail and Distribution Management
Rodríguez-Torrico et al. (2017)^a^	Tell me what they are like and I will tell you where they buy. An analysis of omnichannel consumer behavior	Computers in Human Behavior
Sands et al. (2016)	Segmenting multichannel consumers across search, purchase and after-sales	Journal of Retailing and Consumer Services
Santos and Gonçalves (2019)	Multichannel consumer behaviors in the mobile environment: Using fsQCA and discriminant analysis to understand webrooming motivations	Journal of Business Research
Schneider and Zielke (2020)	Searching offline and buying online – An analysis of showrooming forms and segments	Journal of Retailing and Consumer Services
Schröder and Zaharia (2008)	Linking multi-channel customer behavior with shopping motives: An empirical investigation of a German retailer	Journal of Retailing and Consumer Services
Sebald and Jacob (2020)	What help do you need for your fashion shopping? A typology of curated fashion shoppers based on shopping motivations	European Management Journal
Seock and Norton (2007)	Attitude toward internet web sites, online information search, and channel choices for purchasing	Journal of Fashion Marketing and Management
Shen et al. (2018)	Channel integration quality, perceived fluency and omnichannel service usage: The moderating roles of internal and external usage experience	Decision Support Systems
Singh and Jang (2022)	Search, purchase, and satisfaction in a multiple-channel environment: How have mobile devices changed consumer behaviors?	Journal of Retailing and Consumer Services
Singh and Swait (2017)	Channels for search and purchase: Does mobile Internet matter?	Journal of Retailing and Consumer Services
Sonderegger-Wakolbinger and Stummer (2015)	An agent-based simulation of customer multi-channel choice behavior	Central European Journal of Operations Research
Song et al. (2020)	The Value of Buy‐Online‐and‐Pickup‐in‐Store in Omni‐Channel: Evidence from Customer Usage Data	Production and Operations Management
Sousa et al. (2015)	Customer Use of Virtual Channels in Multichannel Services: Does Type of Activity Matter?	Decision Sciences
Sousa and Voss (2012)	The impacts of e-service quality on customer behaviour in multi-channel e-services	Total Quality Management and Business Excellence
Soysal and Krishnamurthi (2016)	How Does Adoption of the Outlet Channel Impact Customers' Spending in the Retail Stores: Conflict or Synergy?	Management Science
Srisuwan and Barnes (2008)	Predicting online channel use for an online and print magazine: a case study	Internet Research
Sun et al. (2019)	Motivating Effective Mobile App Adoptions: Evidence from a Large-Scale Randomized Field Experiment	Information Systems Research
Sun et al. (2020)	When digitalized customers meet digitalized services: A digitalized social cognitive perspective of omnichannel service usage	International Journal of Information Management
Trenz et al. (2020)	Disentangling the impact of omnichannel integration on consumer behavior in integrated sales channels	MIS Quarterly
Valentini et al. (2011)	Decision Process Evolution in Customer Channel Choice	Journal of Marketing
Valentini et al. (2020)	Identifying omnichannel deal prone segments, their antecedents, and their consequences	Journal of Retailing
van Dijk et al. (2007)^a^	Consumers, channels and communication: Online and offline communication in service consumption	Interacting with Computers
van Nguyen et al. (2022)	Exploring customer experience during channel switching in omnichannel retailing context: A qualitative assessment	Journal of Retailing and Consumer Services
Venkatesan et al. (2007)	Multichannel Shopping: Causes and Consequences	Journal of Marketing
Verhagen and van Dolen (2009)	Online purchase intentions: A multi-channel store image perspective	Information and Management
Verhagen et al. (2019)	The influence of in‐store personnel on online store value: An analogical transfer perspective	Psychology and Marketing
Voorveld et al. (2016)	Consumers' Cross-Channel Use In Online and Offline Purchases	Journal of Advertising Research
Wagner et al. (2020)	Online retailing across e-channels and e-channel touchpoints: Empirical studies of consumer behavior in the multichannel e-commerce environment	Journal of Business Research
Wang et al. (2013)	Understanding the substitution effect between online and traditional channels: Evidence from product attributes perspective	Electronic Markets
Wang et al. (2016)	Understanding multi-channel research shoppers: an analysis of Internet and physical channels	Information Systems and e-Business Management
Wang et al. (2021a)	Contactless channel for shopping and delivery in the context of social distancing in response to COVID-19 pandemic	Electronic Commerce Research and Applications
Xu and Jackson (2019a)	Examining customer channel selection intention in the omni-channel retail environment	International Journal of Production Economics
Xu and Jackson (2019b)	Investigating the influential factors of return channel loyalty in omni-channel retailing	International Journal of Production Economics
Yang et al. (2013)	Why do consumers adopt online channel? An empirical investigation of two channel extension mechanisms	Decision Support Systems
Yang et al. (2014)	Integration and consistency between web and mobile services	Industrial Management and Data Systems
Yang et al. (2007)	Consumers' channel choice for university-licensed products: Exploring factors of consumer acceptance with social identification	Journal of Retailing and Consumer Services
Zhai et al. (2017)^a^	The interactions between e-shopping and store shopping in the shopping process for search goods and experience goods	Transportation

^a^ = articles found by cross-referencing

## Footnotes

1 In addition to the SLRs discussed, there exist several literature reviews on supply chain and logistics aspects of multi- and omnichannel retailing. They include Lafkihi et al. (2019); Melacini et al. (2018); Taylor et al. (2019). For a comprehensive overview on earlier literature reviews on omnichannel retailing, including logistics and supply chain management, see Mishra et al. (2021).

2 All three electronic databases chosen permit the ex-ante filtering of journals by language (= English) and year of publication (= after 2000). We searched the Business Source Complete Collection in EBSCO Host and limited the results to peer reviewed articles available as full text that appeared in academic journals (= source type). In the Scopus database, we searched title, abstract and keywords and limited the results to articles appearing in journals (= source type) and in the subject area business, management and accounting. Finally, we searched the Core Collection in the Web of Science database for title, abstract, author keywords and keywords plus and limited the results to the subject categories of business and management. The literature search took place on May 4, 2022. For ex-post filtering by impact factors, we used the Journal Citation Report (JCR) by Clarivate Analytics 2021 (Journal Citation Reports 2021).

3 Other names for customer needs in the channel choice literature include “shopping motives” (Kim et al. 2019), “goals consumers pursue” (Gensler et al. 2012), and “value-in-use that customers seek in their interactions” (Barwitz and Maas 2018).

4 For a comprehensive overview of the social influence on consumer behavior and, in particular, technology adoption see Graf-Vlachy et al. (2018).

5 This appears to be the case notwithstanding the findings on webrooming cited above; webrooming is a phenomenon associated with channel switching, which remains relatively rare.
